# Association Between Incense Burning and the Risk of Lung Cancer in Asian Population: Meta‐Analysis of Nine Case–Control Studies

**DOI:** 10.1002/cnr2.70095

**Published:** 2024-12-26

**Authors:** Hui‐Wen Tang, Fui‐Ling Voon, Edmund Ui‐Hang Sim

**Affiliations:** ^1^ Faculty of Resource Science and Technology University Malaysia Sarawak Kota Samarahan Malaysia

**Keywords:** Asia, incense, lung cancer, meta‐analysis, smoker

## Abstract

**Background:**

Various studies have explored the potential association between incense burning and the risk of lung cancer. However, the findings from these studies have been inconsistent.

**Objectives:**

This study aimed to provide a more comprehensive understanding of the relationship between incense burning and lung cancer risk in the Asian population through a meta‐analysis.

**Methods:**

This meta‐analysis, which includes nine case–control studies conducted in Asia and identified through Google Scholar, PubMed, and ScienceDirect up to January 7, 2024, was performed to evaluate the relevant literature. Using a fixed‐effects model, the pooled odds ratio (OR) was calculated to determine the overall association between incense burning and lung cancer.

**Results:**

The results of the meta‐analysis revealed a significant association between incense burning and the development of lung cancer (pooled OR = 1.33, 95% confidence interval [CI]: 1.20–1.48). Furthermore, a subgroup analysis was conducted based on smoking status. It was found that ever‐smokers had a significantly higher risk of developing lung cancer when exposed to incense burning (pooled OR = 1.34, 95% CI: 1.09–1.65). Both hospital‐based case–control studies (pooled OR = 1.28, 95% CI: 1.10–1.48) and population‐based case–control studies (pooled OR = 1.39, 95% CI: 1.21–1.60) yielded significant associations between incense burning and lung cancer. Limitations of this study include the lack of detailed histologic information in most of the selected studies, highlighting the need for future research to include cohort studies that can more accurately assess the association between incense smoke inhalation and specific lung cancer subtypes.

**Conclusion:**

In conclusion, the findings of this meta‐analysis, based on nine case–control studies, suggest that the risk of developing lung cancer among Asians may increase with exposure to incense burning.

**Abbreviations**
ALDH1A1: aldehyde dehydrogenase family 1 member A1
AR: androgen receptor
AREG: amphiregulin
AuO: auramine O
CI: confidence interval
DB[*a*,*h*]A: dibenzo[*a*,*h*]anthracene
DHT: dihydrotestosterone
EGFR: epidermal growth factor receptor
F: female
HCC: hospital‐based case–control
HR: hazards ratio
IARC: International Agency for Research on Cancer
M: male
N/A: not applicable
NOS: Newcastle–Ottawa Scale
NSCLC: non‐small cell lung cancer
NTP: National Toxicology Program
OR: odds ratio
PAH: polycyclic aromatic hydrocarbons
PCC: population‐based case–control
PM: particulate matter
ROS: reactive oxygen species
SCLC: small cell lung cancer
TKI: tyrosine kinase inhibitors
VOC: volatile organic compound

## 1 Background

Lung cancer is among the top five diagnosed cancers in Asia and globally. Based on the 2020 GLOBOCAN estimates, Asia accounted for 60% of all newly reported lung cancer cases [1]. Lung cancer is prevalent among Asians, regardless of gender [2]. It is primarily divided into two main histologic classifications: non‐small cell lung cancer (NSCLC) and small cell lung cancer (SCLC). NSCLC is the more common of the two, comprising approximately 80%–85% of lung cancer cases. Within NSCLC, the major subtypes include adenocarcinoma (about 40% of cases), squamous cell carcinoma (25%–30%), and large cell carcinoma (10%–15%) [3]. Adenocarcinoma is typically peripheral and glandular [4, 5], squamous cell carcinoma is usually central and characterized by keratin production [4, 6], and large cell carcinoma is undifferentiated [4, 7, 8]. On the other hand, SCLC accounts for the remaining 15%–20% of lung cancer cases. SCLC is highly aggressive, tends to grow rapidly, and is often associated with smoking [9, 10]. It is centrally located and typically has smaller, flatter cells when compared to NSCLC [11, 12]. Persistent coughing, hemoptysis, unexplainable weight loss, dyspnea, and anorexia are common symptoms of lung cancer [13, 14]. Recognizing these symptoms can lead to an earlier diagnosis and potentially detect the disease at an earlier stage. Another key strategy to reduce the risk of developing lung cancer is to be aware of the potential risk factors. Smoking and second‐hand smoke are identified as major causes of lung cancer [15, 16]. On the other hand, exposure to incense burning is considered a potential risk factor [17, 18, 19].

Incense has been widely used and exposed in Asia [20, 21], especially in East Asia [22]. According to a 2022 survey by the Pew Research Centre, 96% of Cambodians, 92% of Sri Lankans, and 84% of Thais reported using incense [23]. Furthermore, burning incense is a common practice for more than half of the populations of China, India [24], Hong Kong [19], Singapore [25], the United Arab Emirates [26], and 40% of Japan [27]. It has become an integral part of daily life, serving various purposes such as aromatherapy, meditation, and deodorizing [28, 29]. However, it has been reported that incense burns slowly and continuously with incomplete combustion, resulting in the release of smoke containing hazardous substances, including aerosols [30, 31], formaldehyde [30], benzene [28, 32], nitrogen dioxide [33, 34], polycyclic aromatic hydrocarbons (PAHs), significant levels of particulate matter (PM), and various volatile organic compounds (VOCs) [30, 31, 33]. Most of these compounds are known or suspected to promote lung cancer in humans [35, 36]. Numerous case–control studies have examined the impact of incense smoke on the risk of lung cancer, considering inhalation is an avenue of exposure. However, the findings from these studies have been inconsistent. Some studies have found a positive association between incense burning and lung cancer, particularly among the Chinese population in Singapore and individuals who burn incense indoors [37, 38]. On the other hand, research conducted in the Hong Kong population suggested that incense burning is not a significant risk factor for lung cancer in both sexes [39]. Additionally, another study reported no association between incense burning and lung cancer risk, even among smokers [40, 41]. A prospective cohort study conducted by Friborg and colleagues [21] revealed that incense use was not associated with an increased risk of lung cancer, regardless of smoking status. Given these conflicting findings, a meta‐analysis was performed to assess the association between incense burning and lung cancer risk, specifically within the Asian population.

## 2 Methodology

### 2.1 Search Strategy

This meta‐analysis was conducted according to the PRISMA (Preferred Reporting Items for Systematic Reviews and Meta‐Analyses) guidelines [42]. Three electronic databases (Google Scholar, PubMed, and ScienceDirect) were accessed until January 7, 2024 for systematic literature searches. Appropriate keywords (“incense,” “agarbatti,” “bakhoor,” “lung,” “pulmonary,” “cancer,” “carcinoma,” “neoplasm,” and “tumor”) in combination with Boolean operators (“AND” and “OR”) were applied during the literature search. Full texts of all relevant papers were collected, and the reference lists of these papers were reviewed to avoid missing potential articles.

### 2.2 Study Selection

Studies that met all of the following inclusion criteria were included in this meta‐analysis: (i) published in English; (ii) included lung cancer patients; (iii) case–control studies; (iv) conducted on humans; and (v) conducted in Asia. Articles were excluded if they met any of the following exclusion criteria: (i) review articles or letters to the editor; (ii) published in languages other than English; (iii) lacked sufficient information to conduct a meta‐analysis; and (iv) were meta‐analyses.

### 2.3 Quality Assessment

A quality assessment was carried out for each selected study. The quality of case–control studies was evaluated using the Newcastle–Ottawa Scale (NOS) [43]. Studies were categorized as high‐quality, moderate‐quality, and low‐quality based on scores of ≥ 7, 4–6, and ≤ 3, respectively [44, 45]. Studies with low quality were excluded from the meta‐analysis.

### 2.4 Data Extraction

The details listed below were extracted from each of the selected studies: the surname of the first author, year of publication, study population, study design, study period, case–control matching criteria, exposure to incense smoke, number of participants, and the outcome in each group.

### 2.5 Statistical Analysis

The Mantel–Haenszel and inverse variance methods were implemented to evaluate the dichotomous data (ever exposed vs. never exposed). Individuals who were ever exposed to incense burning refer to those who have been exposed or have ever been exposed to incense burning. In contrast, those never exposed are individuals who have never been exposed to incense burning. The results generated an odds ratio (OR) with corresponding 95% CIs and weights for each estimate, which were then visualized in a forest plot. A solid diamond indicated the pooled OR at the bottom of each forest plot. Heterogeneity between the selected studies was measured with Cochran's *Q*‐test and the Higgins *I* ^2^‐test, along with visual inspection of the forest plots [46, 47]. It reflects significant heterogeneity when *p* < 0.05 or I^2^ > 50%. Depending on the degree of heterogeneity, either fixed‐ or random‐effects models will be used to calculate the pooled OR. Since there is no evidence of heterogeneity across the selected studies, fixed‐effect models were applied in all the analyses included in this meta‐analysis [48, 49]. The degree of publication bias was assessed using Egger's test and illustrated with funnel plots [50]. All statistical analyses were performed using R Studio with the meta package (Version 2023.12.0, Build 369) [51, 52].

## 3 Results

### 3.1 Study Selection

**FIGURE 1:**
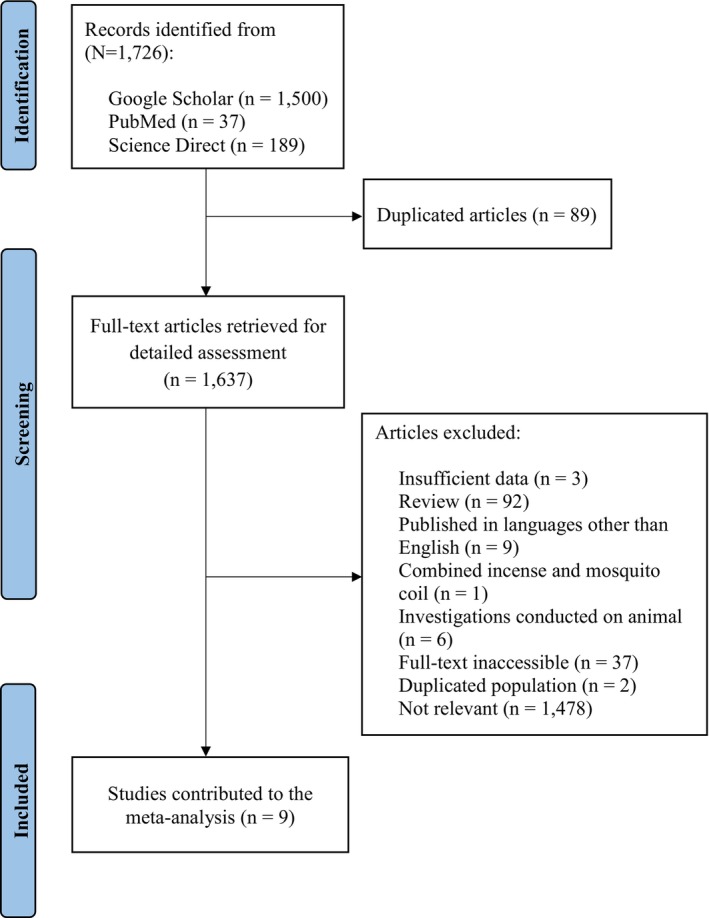
A flow diagram illustrating the process of the studies was chosen to be incorporated into the current meta‐analysis.

Figure 1 shows the flowchart illustrating the process of selecting articles for the study. In summary, 1726 publications were identified through the electronic databases Google Scholar, PubMed, and ScienceDirect. Specifically, 1500 articles were retrieved from Google Scholar, 189 from ScienceDirect, and 37 from PubMed. To conduct a comprehensive review, the remaining 1637 articles were downloaded in full‐text format after removing 89 articles that were found to be duplicates across the three databases. Three publications were excluded from the meta‐analysis due to insufficient data: two were retrieved from Google Scholar [53, 54], and one was found through both Google Scholar and PubMed [21]. Additionally, one paper [18] that examined the combined use of incense and mosquito coils was identified through both Google Scholar and PubMed. Three studies involving animal subjects were obtained from the PubMed database [55, 56, 57], and three from the Google Scholar database [58, 59, 60]. Additionally, 92 review articles did not meet the inclusion criteria, and nine papers were published in languages other than English. Two studies [61, 62] were excluded from the meta‐analysis due to duplicate populations; both were retrieved from the Google Scholar database, while another 37 were not available in full text. Lastly, 1478 studies that were irrelevant or lacked information on exposure to incense burning and lung cancer risk were excluded. The remaining nine studies eligible for the meta‐analysis were sourced as follows: one study from PubMed [37], four from Google Scholar [38, 40, 63, 64], three from both Google Scholar and PubMed [19, 65, 66], and one study from all three databases: Google Scholar, PubMed, and another database [39].

### 3.2 Quality Assessment

The quality of the nine case–control studies was evaluated using the NOS, which covered three main areas: selection, comparability, and exposure. In the selection domain, the evaluation examined whether cases were well‐defined and whether controls were chosen from the same population, thereby ensuring comparability between the groups at baseline. The comparability domain investigated how well the studies controlled for confounding variables and whether cases and controls were matched on relevant factors. Finally, the exposure domain assessed the precision and reliability of the methods used to measure exposure. This comprehensive assessment aimed to ensure that the included studies had reliable procedures and that the findings were supported by high‐quality evidence. Studies were categorized as high‐quality, moderate‐quality, and low‐quality based on scores of ≥ 7, 4–6, and ≤ 3, respectively. The scores are shown in Table 1, which indicates that all the selected studies fell into the moderate‐ or high‐quality category. Among these, seven studies [37, 38, 39, 63, 64, 65, 66], each scoring 7 out of 9, and one study [19] scored 8, were classified as high quality, indicating robust methodological rigor and reliable findings. These high‐quality studies demonstrated clear case definitions, appropriate control selection from the same population, and accurate exposure measurement. The remaining study [40] was classified as moderate quality, reflecting generally sound methodology but with some limitations in areas such as selection assessment. All studies underwent a thorough evaluation to ensure they met the necessary standards for inclusion. Since all selected studies fell into the moderate‐ or high‐quality category, the integrity of the meta‐analysis was maintained, ensuring that the results are based on strong, reliable evidence. This comprehensive selection process aimed to provide a reliable synthesis of the available data, enhancing the validity of the meta‐analytic findings.

**TABLE 1:** Characteristics of studies derived for this meta‐analysis.

Author, year	Study population	Study design	Study period	Gender	collection	Incense burning exposure	Case–control matching criteria	Smoking status	Cases		Controls		Quality assessment
									Total	Female	Total	Female	
Anita et al., 2023	India	HCC	2015–2017	M, F	Interview and questionnaire	Yes^a^ No^b^	Age, gender	N/A	68	N/A	68	N/A	7/9
Chen et al., 2022	Taiwan	HCC	2018–2019	M, F	Face‐to‐face interview	Habitual indoor incense burning (more than 3 days a week for more than 6 months)	Age	N/A	39	N/A	40	N/A	7/9
Phukan et al., 2014	India	PCC	2009–2012	F	Interview	Yes^a^ No^b^	Age, ethnic tribe, gender, geographic	N/A	230	230	460	460	7/9
Tse et al., 2011	Hong Kong	PCC	2004–2006	M	Interview	Never^b^ < 2 times/day ≥ 2 times/day	Age, ethnic, gender, residential district	Never^c^ Ever^d^	1208	N/A	1069	N/A	8/9
Chen et al., 2008	Taiwan	HCC	2002–2004	M, F	Face to face interview	Yes^a^ No^b^	Geographic area	N/A	147	N/A	400	N/A	7/9
Chen et al., 2007	Taiwan	HCC	2002–2006	F	Personal interviews based on a structured questionnaire	Ever^a^ Never^b^	Ethnic, gender	N/A	826	826	531	531	7/9
Chan‐Yeung et al., 2003	Hong Kong	HCC	1999–2001	M, F	Interviewed using a questionnaire by one trained interviewer	No or < 2 years Festival only Daily	Age, ethnic, gender	N/A	331	119	312	113	7/9
Koo et al., 1995	Hong Kong	PCC	1981–1983	F	Personally interview	0 exposure to incense, years 1–39 exposure to incense, years 40–70 exposure to incense, years	Age, gender, housing type, residential district	Never^c^ Ever^d^	189	N/A	197	N/A	6/9
Maclennan et al., 1977	Singapore	HCC	1972–1973	M, F	Interview	Incense burned	Age, dialect, gender	N/A	233	86	300	166	7/9

*Note:* F, female; HCC, hospital‐based case–control; M, male; N/A, not applicable; PCC, population‐based case–control.

^a^ Individuals who have been exposed or have ever been exposed to incense burning.

^b^ Individuals who have never been exposed to incense burning.

^c^ Individuals who have never smoked.

^d^ Individuals who have smoked or have ever smoked.

### 3.3 Study Characteristics

Table 1 summarizes the key characteristics of the nine case–control studies included in this meta‐analysis. It summarize various aspects of each study, such as the author and year of publication, the study population, and the study design. Information on the study period, gender distribution of participants, and data collection methods are also shown in Table 1. Specifics on incense burning exposure and case–control matching criteria were provided, along with smoking status information. Additionally, the table lists the number of cases and controls, including their gender distribution. All nine case–control studies were conducted in Asia and involved two distinct study designs: hospital‐based case–control and population‐based case–control. Data were primarily collected through interviews, with three studies [39, 64, 65] also employing questionnaires. Only two studies [19, 40] provided detailed information on smoking status, which was included in the current meta‐analysis. This comprehensive overview highlighted the diversity in study populations, designs, and methodologies, offering essential context for interpreting the meta‐analysis results.

### 3.4 Incense Burning and Lung Cancer Risk

**FIGURE 2:**
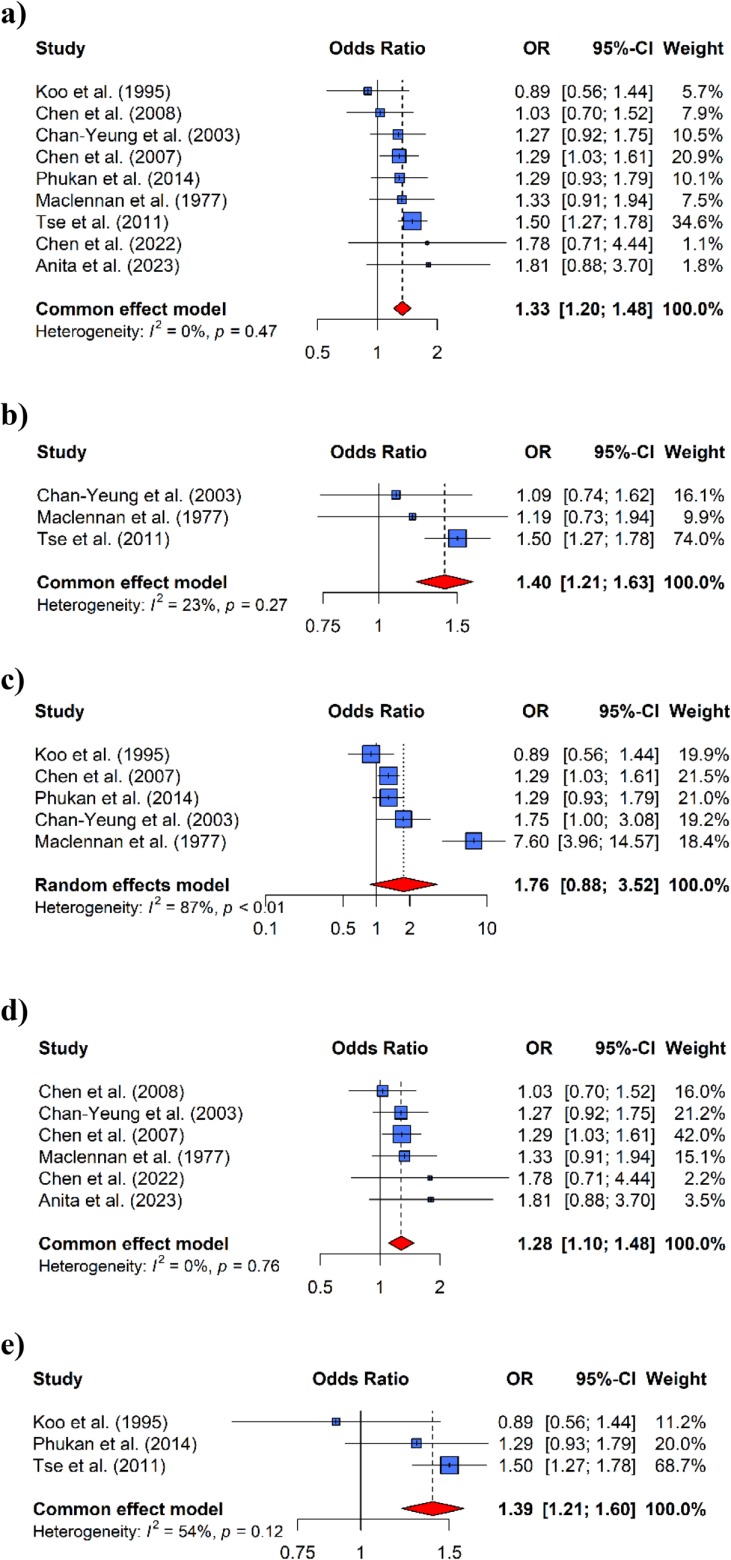
Forest plots were examined to identify the association between exposure to incense burning (never vs. ever) and lung cancer risk. The squares represent the risk estimates specific to each study, with the size of the square reflecting the study's statistical weight, which is the inverse of the variance. The horizontal lines indicate the 95% confidence interval (CI), and the diamond represents the pooled odds ratio (OR) and its corresponding 95% confidence interval. The OR represents the risk of developing lung cancer. (a) Exposed to incense burning versus unexposed. Subgroup analysis is based on (b) males. (c) females. (d) hospital‐based case–control study, and (e) population‐based case–control study.

**FIGURE 3:**
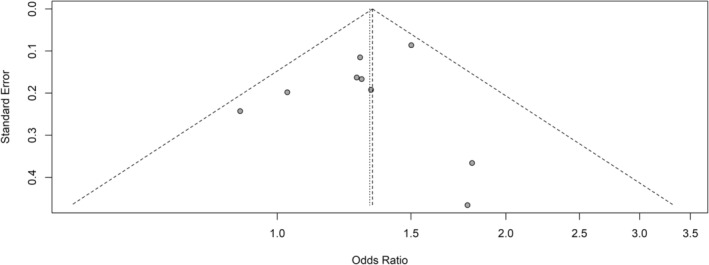
Funnel plot illustrating the publication bias in the studies analyzed in this meta‐analysis. The *X*‐axis represents the odds ratio, and the *Y*‐axis shows the standard error. No indication of publication bias was noted from both the visualization of the funnel plot and Egger's test (*p* = 0.47).

Depending on the heterogeneities, either fixed‐ or random‐effects models were employed to compute the pooled OR for the association between incense burning and the risk of lung cancer. A meta‐analysis was conducted to identify the risk of lung cancer associated with exposure to incense burning, treating it as a dichotomous variable (ever exposed vs. never exposed). The findings are illustrated in Figure 2(a), which revealed that the pooled OR was 1.33 (95% CI: 1.20–1.48) for individuals exposed to incense burning compared to the control group, which was not exposed to incense burning, based on data extracted from the derived nine studies. This pooled OR of 1.33 suggests that individuals exposed to incense burning have a significantly higher chance (33% higher) of developing lung cancer. The Cochran's Q‐test and the Higgins I^2^ test did not reveal significant heterogeneities among the studies [Q = 7.60, *p* = 0.47, $I^{2}$ = 0.00% (95% CI: 0.00%–64.80%), $\tau^{2}$ = 0.00 (95% CI: 0.00–0.10]. Both Egger's test (*p* = 0.47) and the funnel plot visualization (Figure 3) did not provide evidence of publication bias.

### 3.5 Subgroup Analysis

The subgroup analysis by gender is depicted in Figures 2(b) and 2(c). Males had a statistically significant 40% higher risk of developing lung cancer (OR = 1.40, 95% CI: 1.21–1.63), whereas females showed no significant association (OR = 1.76, 95% CI: 0.88–3.52). Additionally, the investigation also explored the association between incense burning and the risk of lung cancer in both ever‐smokers and never‐smokers. The results revealed a significant positive association among ever‐smokers, with a pooled OR of 1.34 (95% CI: 1.09–1.65). However, no significant association was found with never‐smokers, with a pooled OR of 1.04 (95% CI: 0.75–1.44). Moreover, a subgroup analysis was performed based on the sources of subjects' recruitment (hospital‐ and population‐based case–control). The pooled ORs for the hospital‐ and population‐based case–control studies were 1.28 (95% CI: 1.10–1.48) and 1.39 (95% CI: 1.21–1.60), respectively, as shown in Figures 2(d) and 2(e). These findings revealed a positive association between incense burning exposure and the risk of developing lung cancer, with no noticeable heterogeneity among the studies.

## 4 Discussion

The present study highlights a significant association between incense smoke exposure and an increased risk of lung cancer. This finding aligns with broader research on air pollution and its effects on lung cancer, including studies investigating the underlying mechanisms of these associations. Tung and colleagues [67] revealed that while auramine O (AuO), an incense ingredient, did not directly induce malignant transformation, it significantly exacerbated lung cancer malignancy by promoting metastatic abilities and stemness in lung tumor cells. AuO accumulated in the nucleus, induced autophagy, and enhanced the expression of aldehyde dehydrogenase family 1 member A1 (ALDH1A1). Knockdown of ALDH1A1 attenuated AuO–induced autophagy and blocked its promotion of lung tumor malignancy. Additionally, Tu and colleagues [68] demonstrated that incense smoke can sensitize lung cancer cells to epidermal growth factor receptor (EGFR) tyrosine kinase inhibitors (TKIs) by inducing amphiregulin (AREG) expression. AREG, a ligand of the EGFR pathway, plays a critical role in modulating EGFR signaling, which is crucial for cell proliferation and survival. While our study focused on the correlation between incense smoke and lung cancer incidence, the work of Tu et al. suggested that incense smoke influenced lung cancer progression through mechanisms involving EGFR signaling pathways.

Yang and colleagues [69] reported that China, India, and Indonesia are the three Asian nations that account for approximately half of the world's male smokers. More than 40% of men in Eastern Asia (South Korea), Southern Asia (Bangladesh and Maldives), and South‐Eastern Asia (Malaysia and Timor‐Leste) have reported consuming tobacco products [70, 71, 72]. Thus, it is essential to note that the smoking factor confounds the possibility of an association between incense smoke and lung cancer, despite the biologically plausible effects of incense smoke on lung cancer. Cigarette smoking is a well‐known risk factor for lung cancer, and it has been reported that incense burning emits four times as much PM as smoking cigarettes. A meta‐analysis by Kim and co‐authors [73] found that exposure to second‐hand smoke could elevate the risk of lung cancer among individuals who have never smoked. Pollutants released from burning incense are reported to have health effects similar to second‐hand smoke [74]. Furthermore, the toxicity of incense smoke is further increased by several hazardous gases, such as carbon monoxide, carbon dioxide, nitrogen dioxide, and sulfur dioxide, as well as the difficulties associated with disposing of burned incense ash [75].

The stratified analysis based on smoking status in this study revealed a nonsignificant association between incense burning and lung cancer in never‐smokers, but a significant positive relationship in ever‐smokers. However, precautions should be taken when interpreting these results, given that only two studies [19, 40] provided relevant data on smoking status. Gender‐specific subgroup analysis showed that a statistically significant positive association was noted only among males and not females. The observed difference may be attributed to biological variations between males and females, which could lead to varying susceptibility to the carcinogenic effects of incense burning [76, 77]. According to a study by May and colleagues [78], significant differences exist between the immune systems of males and females. For instance, females have been found to exhibit enriched immune gene sets compared to males, especially in the context of lung cancer, as reported by Araujo et al. [79]. Females tend to have stronger innate and adaptive immune responses than males. Furthermore, the androgen receptor (AR), which is primarily expressed in pneumocytes and lung epithelium in male patients [76], plays an essential role in the etiology of lung cancer [80].

**FIGURE 4:**
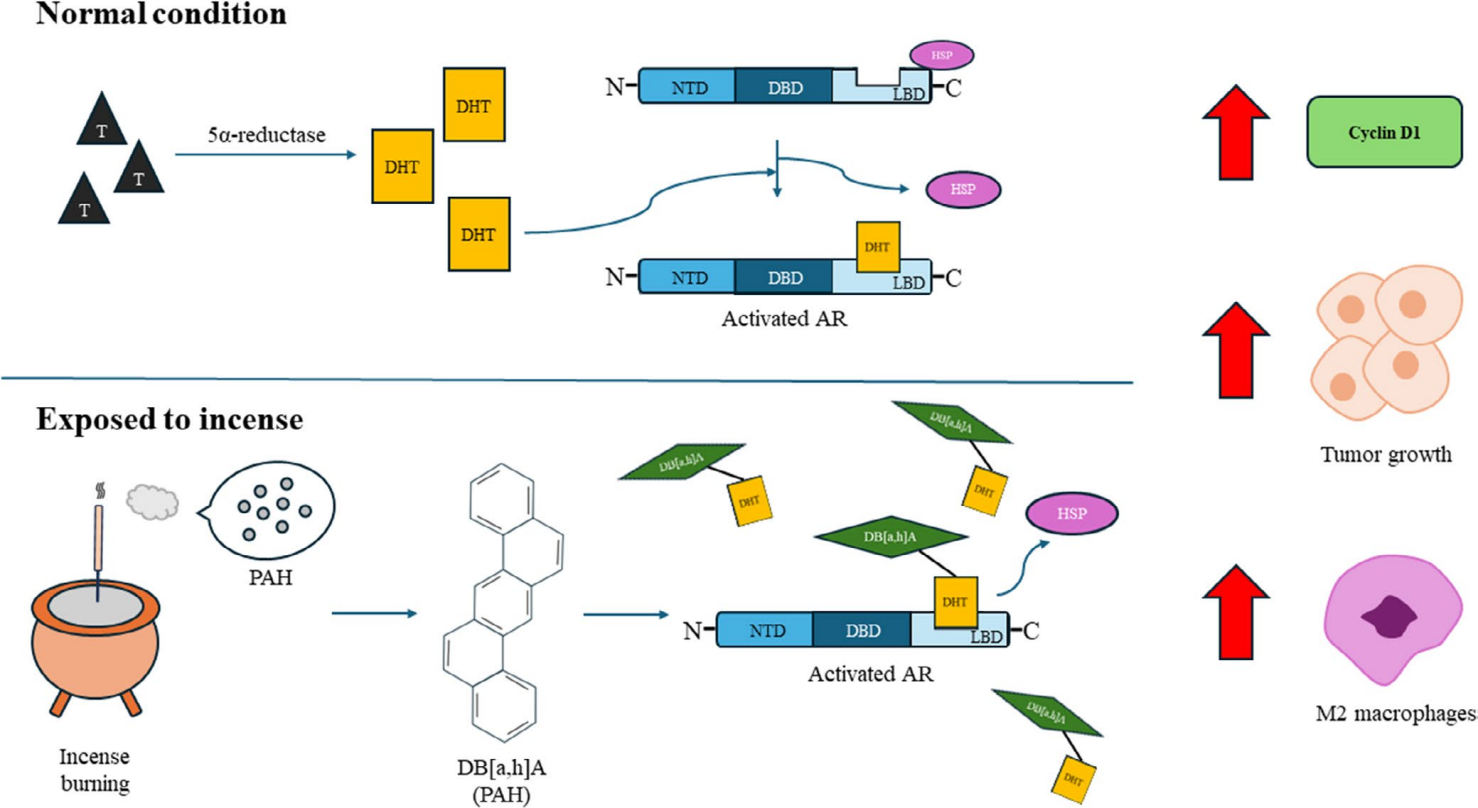
Activation and dysregulation of the androgen receptor (AR) by androgens and environmental pollutants. The AR protein is composed of three fundamental functional domains: the N‐terminal transactivation domain (NTD), the DNA‐binding domain (DBD), and the ligand‐binding domain (LBD) [81, 93]. Androgens, such as testosterone (T), are converted to dihydrotestosterone (DHT) by the enzyme 5α‐reductase. DHT binds to the LBD of AR, leading to the dissociation of heat‐shock proteins (HSPs) and subsequent activation of AR [82, 83, 93]. Incense burning emits PAHs, particularly dibenzo[*a*,*h*]anthracene (DB[*a*,*h*]A) [85], which can provide an additive agonistic effect of androgen in AR activation. As portrayed in the diagram, DB[*a*,*h*]A promotes the increase in AR activation by attaching to DHT and enhances its binding to the LBD of AR [84]. Once activated, AR increases the expression of downstream targets, including cyclin D1 [76, 86], which promotes cell cycle progression and tumor growth [76, 87] and polarization of macrophages toward the tumor‐promoting M2 phenotype [76, 87, 92].

Figure 4 shows that androgens, including testosterone [81, 82, 83], are converted to dihydrotestosterone (DHT) by the enzyme 5α‐reductase. Once formed, DHT binds to the ligand‐binding pocket of the AR, triggering the dissociation of heat‐shock proteins (HSPs) from the AR, which in turn leads to AR activation. A study by Vinggaard and Larsen [84] demonstrated that the PAHs released upon incense combustion, particularly dibenzo[*a*,*h*]anthracene (DB[a,h]A) [85], have an additive effect in enhancing the activation of AR in the presence of androgen. The activation of AR increases the expression of downstream targets like cyclin D1 [76, 86], which drives cell cycle progression and tumor growth [76, 87]. Moreover, pollutants can induce oxidative stress and inflammatory responses [88, 89, 90, 91], further contributing to AR gene mutations and enhancing AR signaling. This process also promotes the polarization of macrophages toward the M2 phenotype [76, 87, 92], which is associated with tumor progression and immune evasion. The differential expression and activation of AR in response to environmental pollutants, the inflammatory microenvironment, and AR gene mutations could contribute to the observed gender disparities in lung cancer incidence and progression.

When subgroup analysis was conducted by sources of subjects' recruitment, it was found that both hospital‐ and population‐based case–control studies showed statistically significant positive associations. This indicates that regardless of whether the subjects were recruited from hospitals, communities, or any other setting, there was a significant association between incense burning and the risk of lung cancer. Furthermore, the consistency of these associations across multiple recruitment sources suggests that the relationship is not confined to a specific population or setting. This strengthens the generalizability of the findings. Therefore, the subgroup analysis based on the sources of subject recruitment enhances the overall meta‐analysis by providing an additional perspective on the association between incense burning and lung cancer risk in various communities.

Despite understanding the exposure of incense burning and its associations with lung cancer through this meta‐analysis, the histology of lung cancer remains inadequately addressed. Although a subgroup analysis focusing on lung cancer histology was initially planned to explore associations with incense burning, data limitations precluded this analysis. However, the literature suggests that NSCLC, particularly adenocarcinoma, may be more closely associated with incense smoke and other environmental exposures. This hypothesis aligns with previous studies linking adenocarcinoma to air pollution [94, 95, 96]. Adenocarcinoma, the most common subtype of NSCLC, originates in the glandular cells of the lung and is often found in peripheral regions [4, 5], making it particularly vulnerable to inhaled toxins. The current literature indicates that environmental factors, such as incense smoke, may differentially impact various lung cancer subtypes, underscoring the need for targeted research. As a result, this finding summarizes the general associations between lung cancer and incense burning without specifically focusing on histologic subtypes. Future research should address this gap by incorporating detailed histological data and exploring the differential effects of environmental exposures on various lung cancer subtypes to enhance our understanding and risk assessment of these associations.

It is crucial to acknowledge several limitations of this meta‐analysis. First, it is worth noting that this meta‐analysis solely included observational studies. Even with strict controls, observational studies are susceptible to various biases, such as recall or information bias. Second, the “ever or never” categorization of incense burning exposure in the nine case–control studies disregards the frequency or quantity of incense burned daily, weekly, or monthly. This limitation restricts the analysis of the association between exposure to incense burning and the risk of lung cancer. Third, the relevant data extracted from the included studies spanned 46 years, from 1977 to 2023, due to the limited availability of recent data. Consequently, the accuracy of the results with current incense products may be questionable. Fourth, it is important to note that this meta‐analysis relied on data extracted from only nine studies. Therefore, the possibility of publication bias cannot be ruled out, despite the absence of any indication of publication bias according to Egger's test and the funnel plot visualization. Furthermore, incorporating diverse hospital‐ and population‐based case–control studies increased the variability among study populations. While this broadens the generalizability of the findings, it also introduces variability that may reduce the precision of the meta‐analysis results. Besides, with only two out of nine studies providing relevant data on smoking status, the meta‐analysis may have reduced statistical power to detect genuine associations or differences between incense burning and the risk of lung cancer based on smoking status.

Given the overall association between incense burning and increased lung cancer risk, this study highlights the importance of exploring how such environmental exposures may influence lung cancer more broadly. Notably, out of the nine selected studies, only one [37] reported the histology of lung cancer, identifying adenocarcinoma, while the remaining eight lacked relevant histologic information. This limitation reveals a significant gap in the current research, particularly the glaring absence of prospective or retrospective cohort studies, which are essential to complement the existing case–control investigations. Future research should focus on filling this gap by conducting cohort studies that can provide more robust and detailed insights into the association between incense smoke inhalation and specific lung cancer subtypes. Such studies will be crucial for improving the accuracy of risk assessments and gaining a comprehensive understanding of how environmental exposures, such as incense smoke, may differentially impact various forms of lung cancer, particularly NSCLC subtypes. Additionally, to enhance the reliability of findings, future research should incorporate more objective measures, such as environmental monitoring or biomarker analysis, to substantiate and complement interview data.

## 5 Conclusions

In conclusion, the present meta‐analysis, comprising nine case–control studies, suggests that the risk of developing lung cancer among Asians may increase when they are exposed to incense burning. According to the gender‐specific subgroup analysis, males have a higher probability of developing lung cancer compared to females. Additionally, the subgroup analysis based on smoking status demonstrates that ever‐smokers are at a greater risk of developing lung cancer compared to those who have never smoked. To establish a more definitive conclusion, future studies should prioritize investigating the association between incense burning and the risk of lung cancer, particularly in more recent periods, to enhance our current understanding of this potential health concern. Furthermore, it is imperative to conduct further research to explore the types of lung cancer susceptible to this association, evaluate causality, and validate the findings in diverse populations.

## Author Contributions

**Hui‐Wen Tang:** conceptualization (equal), formal analysis (lead), validation (equal), visualization (lead), writing – original draft (lead), writing – review and editing (equal). **Fui‐Ling Voon:** conceptualization (equal), formal analysis (supporting), supervision (supporting), validation (supporting), writing – review and editing (equal). **Edmund Ui‐Hang Sim:** conceptualization (equal), formal analysis (supporting), supervision (lead), validation (lead), writing – review and editing (equal).

## Ethics Statement

The authors have nothing to report.

## Consent

The authors have nothing to report.

## Conflicts of Interest

The authors declare no conflicts of interest.

## Data Availability

The data that support the findings of this study are available from the corresponding author upon reasonable request.

## Appendix A

### A.1

Database: Google Scholar

**Table:**

Search words	Limits (filter, limits, and refine)	Number of records
“incense” **AND** “lung cancer” ‐”mice” ‐”rat”	Publication years: 2013–2023	1820
“incense” **AND** “lung cancer” ‐”mice” ‐”rat”	Publication years: 2008–2012	435
“incense” **AND** “lung cancer” ‐”mice” ‐”rat”	Published until year 2007	591

Database: ScienceDirect

**Table:**

Search words	Limits (filter, limits, and refine)	Number of records
“incense” **AND** “lung cancer”	Publication years: 2013–2023 Article type: Research articles	163
“incense” **AND** “lung cancer”	Publication years: 2008–2012 Article type: Research articles	19
(Agarbatti **OR** Bakhoor **OR** incense) **AND** (lung **OR** pulmonary) **AND** (cancer **OR** carcinoma **OR** neoplasm **OR** tumour)	Publication years: 1700–2007 Article type: Research articles	95

Database: PubMed

**Table:**

Search words	Limits (filter, limits, and refine)	Number of records
“incense” **AND** “lung cancer”	Publication years: 2013–2023	6
“incense” **AND** “lung cancer”	Publication years: 2008–2012	4
(Agarbatti **OR** Bakhoor **OR** incense) **AND** (lung **OR** pulmonary) **AND** (cancer **OR** carcinoma **OR** neoplasm **OR** tumour)	Publication years: 2013–2023	14
(Agarbatti **OR** Bakhoor **OR** incense) **AND** (lung **OR** pulmonary) **AND** (cancer **OR** carcinoma **OR** neoplasm **OR** tumour)	Publication years: 2008–2012	5
(Agarbatti **OR** Bakhoor **OR** incense) **AND** (lung **OR** pulmonary) **AND** (cancer **OR** carcinoma **OR** neoplasm **OR** tumour)	Publication years: 1990–2007	8
